# Not too quick on “Debunking the myth of ‘Blue Mondays’”

**DOI:** 10.1177/02698811221100713

**Published:** 2022-08-04

**Authors:** Jacob Flameling, Floor van der Does, Nancy van Veelen, Eric Vermetten

**Affiliations:** 1 Leiden University, Leiden, Zuid-Holland, The Netherlands; 2 Leiden University Medical Center, Leiden, Zuid-Holland, The Netherlands

Dear Editor,

In their recent article “Debunking the myth of ‘Blue Mondays’: No evidence of affect drop after taking clinical MDMA,” Sessa et al. (2021) reported on results from an open-label study researching the potential use of 3,4-Methylenedioxymethamphetamine (MDMA) in the treatment of alcohol use disorder (AUD). Although we applaud the efforts to ascertain the safety and efficacy of clinical MDMA-assisted therapy for mental health conditions, we have serious concerns that the claims made in this article are not justified by the data. The main conclusion of the authors is that “there is no observable decline in mood after controlled dosing of MDMA in clinical settings,” thereby suggesting “that the ‘comedowns’ previously associated with the substance may be explained by confounds in research relating to the illicit sourcing of the drug and specific environmental setting for recreational consumption.” Although we see the theoretical merit of this claim, and do consider it plausible that sleep disturbances and poor self-care may contribute to the “Blue Monday” effect, we believe that the conclusion that the myth of “Blue Mondays” is debunked is not justified.

## Insufficient power and methodology

First and foremost, the title of the article, “Debunking the myth. . .” suggests that there is enough evidence to assume that the “Blue Monday” effect after taking clinical MDMA is a myth, and that it has hereby been debunked. However, the authors must be aware that limited sample size (*N* = 14) means that null-findings in this study could easily be a function of a lack of statistical power or a coincidence. Moreover, the problem of limited power is exacerbated by choices made in the statistical analysis of the data. Most patients had multiple MDMA sessions, resulting in a cumulative number of 26 sessions. In order to deal with the interdependency of the data acquired from different sessions in the same participant, the authors have decided to average these POMS scores to result in a single list of 7 POMS scores per patient. However, this means that valuable information is lost as the number of data points in the study is almost halved. This is especially pertinent since an earlier study on the same dataset (see Figure 4 in Sessa et al. (2021)) shown that the course of mood scores differs after the first and second sessions. The correct way to deal with these nested data would have been to use a hierarchical model. We encourage the authors to perform a reanalysis of their data using the correct statistical procedures, which could enhance the credibility of their article.

The authors stated that “the study was adequately powered to detect improvements in sleep quality as well as mood based on recreational studies with MDMA (Parrott and Lasky, 1998).” Rather than citing a study with a similar *N*, a power analysis would have been preferable.

Additionally, using this ANOVA, the authors assessed whether a significant difference in mood score occurs in the 7 days after the dosing session. They detected no significant changes, and concluded that there is no evidence of an affect drop after taking clinical MDMA. It would have been informative to know whether this was true for all participants at all respective sessions, as only effects on the group level are reported. Furthermore, the authors posited that the positive mood exhibited by the participants in the 7 days after the session was indicative of an “afterglow” effect. Because there is no control group or baseline measurement of the profile of mood states (POMS), this is a conclusion that cannot be drawn on the basis of these data. As there is nothing to compare these scores with, it cannot be stated that mood was lifted after the session, and this supposed lift in affect was an MDMA effect. It is even possible that mood was more negative after the MDMA session than before—as there is no baseline measurement, we simply do not know. The only fair conclusion that can be drawn is that on the group level, no significant differences could be detected in mood scores of these 14 patients in the 7 days following MDMA dosing sessions.

Lastly, in their discussion, the authors fail to cite the studies from Liechti et al. (2001) and Vizeli and Liechti (2017), which do find evidence for a mood drop in the days following clinical MDMA intake, thereby presenting an unrepresentative view of the current literature.

## Lack of evidence of a causal role of MDMA in improved sleep quality

The authors reported that compared to baseline, patients’ quality of sleep improved at the 3 months and 6 months follow-up. We have two concerns about this analysis. First, the lack of a control group means these findings cannot be attributed to the effects of MDMA, and could also be caused by non-specific effects of therapy. Second, in the introduction of the article, the authors did not explain why they measured sleep quality months after MDMA administration. The authors’ only mention of sleep quality in the introduction is their hypothesis that the “Blue Monday” effect may be partially due to a lack of sleep, exhaustion, and interactions with other psychoactive drugs, typical for recreational use of MDMA. We, therefore, are left to wonder why sleep quality as measured by the Pittsburg Sleep Quality Index 3 and 6 months after the sessions were reported, even though sleep quality was also measured during the 7 days after MDMA administration using the Leeds Evaluation Questionnaire. In the context of this article, it would make more sense to report on the latter sleep scores.

## Social desirability bias in reporting cravings and use of “illicit” MDMA

The authors reported that no participants reported to have “taken illicit MDMA or Ecstacy” nor “had any desire to take illicit MDMA or Ecstasy.” We wonder if the authors have considered that the use of the word “illicit” may have implied to the participants that this was an undesirable outcome, thereby increasing the likelihood of a socially desirable “No” response.

## Ambiguity in reporting of anecdotal responses

In the qualitative section of this article, the authors decided to only include “all responses that were judged to be clear and unambiguous.” We have several questions about this decision. For instance, how did the authors decide what responses were “clear and unambiguous”? Were there multiple raters, and can the authors report inter-rater reliability? These questions also apply to the “list of representative questions and responses” included in Table 3. What does representative mean in this case, and how was representativeness assessed? Additionally, it is possible that rates of unclear and ambiguous responses change following MDMA sessions. Therefore, if these responses are thrown out, valuable information may be lost. Although we find the quotes in Table 3 inspiring to read, they would be more informative if these issues were cleared up. We would encourage the authors to publish all questions and responses as Supplementary Material so that the reader can judge the representativeness and ambiguity of the responses themself.

## Failure to correct for multiple comparisons

According to the pre-registration of this study, 23 secondary outcome measures were assessed. We wonder why the authors did not correct for multiple testing, and why they did not justify their decision not to. We expect to see more articles coming from this study, and hope to see this issue addressed.

We agree with the authors that the use of psychedelic-assisted therapy in the treatment of various psychiatric disorders shows great promise. Although public opinion of these compounds is improving, many patients still have concerns. We applaud the authors’ effort to ease these concerns, and their attempts to support this with research data. However, we think it does injustice to this newly emerging field to believe that the data used in this study are sufficient to substantiate the claims in the title and conclusion. As the authors are operating in a field that is the object of significant public attention and scrutiny, and which may present a source of renewed hope for patients who did not benefit from currently approved treatments, it is very important that the methodologies and statistical and causal inference presented in scientific articles are sound. “Debunking the myth of ‘Blue Mondays’” is a compelling title, but by boldly overstating their case, the authors failed to achieve its premise.

## References

[bibr1-02698811221100713] Liechti ME Gamma A Vollenweider FX (2001) Gender differences in the subjective effects of MDMA. Psychopharmacology 154: 161–168. 11314678 10.1007/s002130000648

[bibr2-02698811221100713] Sessa B Aday JS O’Brien S , et al. (2021) Debunking the myth of ‘Blue Mondays’: No evidence of affect drop after taking clinical MDMA. J Psychopharmacol 36: 360–367. 34894842 10.1177/02698811211055809

[bibr3-02698811221100713] Vizeli P Liechti ME (2017) Safety pharmacology of acute MDMA administration in healthy subjects. J Psychopharmacol 31: 576–588. 28443695 10.1177/0269881117691569

