# Supplementary material for: Not too quick on “Debunking the myth of ‘Blue Mondays’”
Source: J Psychopharmacol. 2022 Aug 4;36(8):1001–4. doi: 10.1177/02698811221100713 (PMC9354057; doi:10.1177/02698811221100713)
Supplement: Related texts

# Letter: Reply to: Not too quick on “Debunking the myth of ‘Blue Mondays’”

**Published:** 2022-08-04

Dear Flameling et al.,

Thank you for your kind comments applauding our efforts to ascertain the safety and efficacy of clinical 3,4-methylenedioxymethamphetamine (MDMA)-assisted therapy for mental health conditions. We agree that this is an important topic. The focus of our article (Sessa et al., 2022) was to highlight the differences between safety and risk issues seen in recreational ecstasy use compared to data emerging from the clinical use of MDMA. Looking at the list of authors of our article, you will see we sought multiple inputs from different disciplines within the fields of clinical MDMA and recreational ecstasy experts to provide an informed and wide-reaching opinion on the issue.

In general, the Blue Mondays’ article has received positive support. But it is certainly the case that the choice of the article’s title (“Debunking the myth of ‘Blue Mondays’*”*) has fostered some criticism. This is largely based on a misunderstanding on the part of some critics who did not read the article in full—and assumed we were suggesting that subjective reports of affect drop post-ecstasy use do not occur amongst recreational users. Although our message was quite the contrary, the phenomenon of post-ecstasy affect drops is a widely reported experience, and the purpose of the article was to contrast this phenomenon with what we saw in our prospective, clinical MDMA study. Although these nuances are difficult if not impossible to fully capture in an article title, we acknowledge that using less powerful language, such as “Challenging the narrative” rather than “Debunking the myth,” may have led to less confusion among readers and better reflected the level of empirical evidence presented in the article.

We shall address all the points you raised in your letter below:

## Insufficient power and methodology

In respect of your comments about power and methodology, as above, we were *not* stating that Blue Mondays do not exist in recreational user populations. Quite the contrary, they do. In respect of power: across 26 clinical MDMA sessions, we did not elicit one single report of acute comedowns. All participants reported no negative disturbance to affect at the end of the day after taking MDMA as the drug wore off. No comedowns. This is a highly significant outcome over 26 separate sessions with clinical MDMA. Although we agree that a power analysis would have been preferable, the study was mandated to use a small sample size given it was an exploratory trial of MDMA therapy for alcohol use disorder (AUD).

Thank you for your suggestion regarding re-analyzing the data using alternative statistical methods. We will take this into consideration. We chose to use ANOVA testing to approximate the methods used in influential recreational MDMA studies that found mood drops post-dosing (e.g., Parrott and Lasky, 1998, which at time of writing had >450 citations). Additionally, your suggestion that we include citations to Liechti et al. (2001) as well as Vizeli and Liechti (2017) is valuable, and we appreciate you directing us to this work. It is worthy of note, that Vizeli and Liechti (2017), also using ANOVA testing, found “These safety data do not raise any concerns related to further studies of MDMA as an adjunct to psychotherapy in controlled medical environments,” and that “the risks and benefits of using MDMA in patients with psychiatric disorders need further study.”

## Lack of evidence of a causal role of MDMA in improved sleep quality

Regarding your commentary about a lack of a control group to accurately test whether reported improvements in sleep are specifically due to MDMA drug effects or rather may be attributed to the psychotherapy, indeed. As stated, this is an open-label study, so all outcomes must be interpreted as such. Only a double-blind, placebo-controlled RCT could separate active drug effects from treatment-nonspecific effects (see Aday et al., 2022). We are currently planning such a study.

The Pittsburg Sleep Quality Index (PSQI) is well-suited for use in studies where analysis of sleep quality is not the primary outcome, due to its brevity and returning of a single score representing overall sleep quality (Faulkner and Sidey-Gibbons, 2019). The Leeds Sleep Questionnaire (LSQ) was also conducted as part of the Bristol Imperial MDMA in Alcoholism (BIMA) study, however, was not included in this publication as the PSQI was more succinct in its findings, and sleep was not the primary focus. Future publications will consider as of yet unpublished measures where appropriate.

## Social desirability bias in reporting cravings and use of “illicit” MDMA

In respect to our question regarding participants’ use of non-clinical/illicit/illegal MDMA/ecstasy, this was a necessary question to include given that the study is exploring safety and tolerability. The question was asked—and where necessary was discussed face to face—openly with participant responders. You state that using the word “illicit” could have implied to the participants that this was an undesirable outcome, thereby increasing the likelihood of a socially desirable “No” response. It is important to clarify that the study team had a positive therapeutic relationship with all the participants, and we have no reason to suspect that participants would have been disingenuous in answering this question. Almost all of the participants had a history of substantial past polydrug use before coming into the study with AUD and had been frank and open about this at screening interviews. Therefore, the likelihood that this sample would consider admission of illicit MDMA to be “socially undesirable” would, in our opinion, be low.

## Ambiguity in reporting of anecdotal responses

In this article, we have not attempted to carry out a formal qualitative analysis of participants’ verbatim reports of tolerability of the study. Rather, we included some participant quotes to provide some human context to the study. Formal qualitative analysis is indeed a complex skill that requires systematic analysis of participant reports; we are explicit in the article that these are a collection of “anecdotal reports,” and nowhere do we claim to have conducted a qualitative analysis. A more-detailed reporting and analysis of all patients’ comments is beyond the scope of the article. However, we agree that qualitative analyses should be increasingly considered when designing clinical trials with psychedelics to identify mechanisms, risks, and benefits that may not be captured by psychometric scales chosen a priori.

## Failure to correct for multiple comparisons

This is so far the third article to be published that came out of the full BIMA project. We did indeed take many more outcome measures than have currently been published, and we are planning to submit further articles for publication. We plan an article soon that will explore the therapeutic psychological model employed in the study, which will include results of several unpublished measures, including commentary about participants’ trauma histories and changes in their compassion and empathy throughout the study. Although the criticism regarding failure to correct for multiple corrections is well-intentioned, there are a number of practical considerations that limit this technique’s use, particularly in small, exploratory studies (Althouse, 2016). Additionally, in this instance, because examining post-acute mood was decided a priori and an important aspect of the study design, it was justifiable to not adjust *p*-values related to mood.

## Conclusion

We welcome debate about our interesting findings. We feel our recent Blue Mondays article contributes positively to the field by providing a clear report of the relative lack of adverse effects seen with clinical MDMA administration in contrast with the widely reported negative anecdotes seen with recreational use (e.g., comedowns and post-ecstasy affect drop). This is especially relevant given the fact that we were studying potentially vulnerable patients with significant mental and physical illness. We appreciate the criticisms about the article’s hard-hitting title, which has certainly resulted in considerable debate. We hope this discourse can ultimately facilitate further widescale discussions about this important topic and lead to a more nuanced understanding of the risks and benefits of MDMA.

Yours Sincerely,



**Dr. Ben Sessa**

**Dr. Jacob S. Aday**

**Steve O’Brien, BSc**

**Dr. H. Valerie Curran**

**Professor Fiona Measham**

**Dr. Laurie Higbed**

**Professor David J. Nutt**



## References

[bibr4-02698811221100713] AdayJS HeifetsBD PratscherSD , et al. (2022) Great expectations: Recommendations for improving the methodological rigor of psychedelic clinical trials. Psychopharmacology 239: 1989–2010.3535915910.1007/s00213-022-06123-7PMC10184717

[bibr5-02698811221100713] AlthouseAD (2016) Adjust for multiple comparisons? It’s not that simple. Ann Thorac Surg 101: 1644–1645.2710641210.1016/j.athoracsur.2015.11.024

[bibr6-02698811221100713] FaulknerS Sidey-GibbonsC (2019) Use of the Pittsburgh sleep quality index in people with schizophrenia spectrum disorders: A mixed methods study. Front Psychiatry 10: 284.3114313110.3389/fpsyt.2019.00284PMC6520598

[bibr7-02698811221100713] LiechtiME GammaA VollenweiderFX (2001) Gender differences in the subjective effects of MDMA. Psychopharmacology 154: 161–168.1131467810.1007/s002130000648

[bibr8-02698811221100713] ParrottAC LaskyJ (1998) Ecstasy (MDMA) effects upon mood and cognition: before, during and after a Saturday night dance. Psychopharmacology 139: 261–268.978408310.1007/s002130050714

[bibr9-02698811221100713] SessaB AdayJS O’BrienS , et al. (2022) Debunking the myth of ‘Blue Mondays’: No evidence of affect drop after taking clinical MDMA. J Psychopharmacol 36: 360–367.3489484210.1177/02698811211055809

[bibr10-02698811221100713] VizeliP LiechtiME (2017) Safety pharmacology of acute MDMA administration in healthy subjects. J Psychopharmacol 31: 576–588.2844369510.1177/0269881117691569
